# New aspects in the pathogenesis and management of subacute thyroiditis

**DOI:** 10.1007/s11154-021-09648-y

**Published:** 2021-05-05

**Authors:** Magdalena Stasiak, Andrzej Lewiński

**Affiliations:** 1 Department of Endocrinology and Metabolic Diseases, Polish Mother’s Memorial Hospital, Research Institute, 281/289 Rzgowska St, 93-338 Lodz, Poland; 2 Department of Endocrinology and Metabolic Diseases, Medical University of Lodz, 281/289 Rzgowska St., 93-338 Lodz, Poland

**Keywords:** Subacute thyroiditis, *HLA*, Recurrence, Clinical course, SARS-CoV-2, COVID-19

## Abstract

Subacute thyroiditis (SAT) is a thyroid inflammatory disease, whose pathogenesis and determinants of the clinical course were unclear for many decades. The last few years have brought many clinically significant new data on the epidemiology, pathogenesis and management of SAT. Several human leukocyte antigen (HLA) alleles were demonstrated not only to increase the risk of SAT, but also to correlate with SAT clinical course and determine the risk of recurrence. The world-wide epidemic of the coronavirus disease 19 (COVID-19) has provided new observations that the severe acute respiratory syndrome-coronavirus-2 (SARS-CoV-2) can be a potent SAT-triggering factor, and that the clinical course of SAT in patients affected by COVID-19 is different from a typical one. Additionally, many new trends in the clinical course are emerging. In the last years, painless course of SAT is more and more often described, constituting a special challenge in patients hospitalized due to COVID-19. Despite an excellent availability of diagnostic methods, several difficulties in SAT differential diagnosis can be currently encountered and the proper diagnosis and treatment is frequently delayed. False positive diagnoses of SAT in patients with malignancies of poor prognosis constitute a life-threatening problem. Taking into account all the new aspects of SAT pathogenesis and of its clinical course, the new – modified – SAT diagnosis criteria have been proposed.

**Supplementary Information:**

The online version contains supplementary material available at 10.1007/s11154-021-09648-y.

## Introduction

Subacute thyroiditis (SAT) (also called granulomatous thyroiditis or de Quervain`s thyroiditis) is a thyroid inflammatory disease, whose pathogenesis and determinants of the clinical course were unclear for many decades. The last few years have brought many clinically significant achievements in this field. Additionally, the clinical course of SAT was demonstrated to evolve in time, and some features considered previously as virtually pathognomonic cannot be considered such any more. Moreover, the new coronavirus disease 19 (COVID-19) pandemic has been spread worldwide since autumn 2019. As the pandemic has been progressing, more and more evidence has emerged on direct correlation between the severe acute respiratory syndrome-coronavirus-2 (SARS-CoV-2) infection and SAT, as well as on specific clinical features of SAT characteristic for the new triggering factor. The data from studies published in the recent few years shed exceptionally significant new light on epidemiology, pathogenesis, and management of SAT, and essentially changed the previous state-of-art. Therefore, the aim of this review is to summarize the most recent advances in SAT, with special attention given to the clinical application of the novel findings.

Study selection flowchart is available as a Supplementary material (Fig. S1).

## Pathogenesis

### Genetic background

Susceptibility to SAT has been considered to be associated with the occurrence of certain types of human leukocyte antigens (HLA) since 1975. In that year, Nyulassy et al. [1] first reported the increased frequency of HLA-B*35 in patients with SAT. Since then, the significant correlation between SAT and HLA-B*35 has been confirmed in several different populations [2–6]. The presence of HLA-B*35 was observed in up to 70% of SAT patients [2, 7], but it was considered doubtful that HLA-B*35-negative patients had virtually no genetic background of SAT. Thus, in the meantime, some authors analyzed other potential associations between HLA and SAT, but their results were highly inconsistent and did not provide reliable conclusions [7–9]. Those authors applied much older, especially serological methods whose accuracy is substantially lower than that of the recently used high resolution method. Recent improvement of diagnostic methods brought the novel insight into the genetic background. In 2020, our research team demonstrated the new association between HLA and SAT in the Caucasian population [10]. In addition to the previously described correlation with HLA-B*35, SAT was proved to be associated with the presence of HLA-B*18:01, -DRB1*01 and -C*04:01 [10]. In Caucasians, HLA-C*04:01 is in a linkage disequilibrium with HLA-B*35:01/02/03, i.e. these two alleles commonly occur together because of the close location of their loci [11]. Therefore, HLA-C*04:01 alone cannot be treated as an independent SAT risk factor due to its linkage with the previously described HLA-B*35. However, the presence of any of the two haplotypes should be considered as a marker of genetic susceptibility to SAT [10]. No such linkage disequilibrium has been described for HLA-B*18:01 or -DRB1*01 so these alleles should be considered new, completely independent SAT risk factors. The study provided the set of four alleles whose assessment allows confirming the genetic predisposition for SAT in almost all patients [10].

As the studies on the novel genetic background of SAT were progressing, the new data on the influence of the HLA alleles on the clinical course of SAT were emerging. Currently, several new publications on this important issue are available and are discussed below.

### Triggering factors

Previous viral infection (occurring approximately 2–6 weeks earlier) is considered a SAT triggering factor in genetically predisposed individuals. The types of viruses associated with the occurrence of SAT include Coxsackie viruses, Echo viruses, adenoviruses, influenza viruses, mumps and rubella viruses, parvovirus B19, orthomyxovirus, HIV, Epstein-Barr virus, hepatitis E and measles virus [12, 13]. A few cases of SAT associated with dengue virus infection have recently been described [14]. During the last year, we have been dealing with the SARS-CoV-2 pandemic spreading around the world. This RNA virus causes COVID-19. So far, over 100 million people have been affected around the world, and this number will be undoubtedly increasing further. Prior to the current pandemic, no association of coronaviruses with the development of SAT was reported [12]. Observations on the pre-existing SARS-CoV-1 virus indicated possible damage to thyroid follicular cells and subsequent fibrosis, which corresponded to destructive thyroiditis [15]. The development of thyroid diseases in the course of severe acute respiratory syndrome (SARS) has been associated with various mechanisms of thyroid damage, including excessive immune response, immunodeficiency associated with infection, or direct cellular damage [16]. SARS-CoV-2 exhibits significant tissue tropism, including the high affinity to the thyroid tissue. The key factor in SARS-CoV-2 infection is an angiotensin converting enzyme 2 (ACE2), which serves as a 'receptor' enabling virus to enter the cell. Thyroid cells are rich in ACE2 [17–19]. Rotondi et al. has recently demonstrated that the mRNA encoding the ACE-2 receptor is highly expressed in thyroid follicular cells, making them a potential target for SARS-CoV-2 entry [20]. Due to the increased affinity of SARS-CoV-2 to the thyroid gland, attention was drawn to the potential risk of developing several potential forms of thyroiditis. Actually, some cases of Graves' disease (GD) [21] and Hashimoto's thyroiditis [16] have been described, but SAT appeared to be the most common COVID-19-related thyroiditis [19]. Despite the very recent onset of the COVID-19 pandemic, many cases of SAT in direct relation to SARS-CoV-2 infection have already been described. Thus, SARS-CoV-2 should be included into the list of viral triggering factors of SAT. Moreover, as the pandemic is still progressing, we should consider SARS-CoV-2 as the most important triggering factor of SAT at the present time.

Very interesting observation was published by Nakagawa et al. [22] who reported SAT in 71-year-old Japanese female with psoriatic arthritis treated initially with secukinumab, an interleukin (IL)-17A inhibitor. Due to treatment failure, the therapy was changed to adalimumab (ADA), a tumor necrosis factor (TNF)-α inhibitor, which triggered SAT occurrence. Treatment was again modified to ixekizumab, an IL-17A inhibitor, without SAT relapse [22]. This very recent case constitutes a confirmation of previous reports on SAT developing during treatment with TNF inhibitors. Those cases include patients with various rheumatic diseases treated with such TNF inhibitors as ADA, etanercept, and infliximab [23–25]. The time lag from the beginning of the therapy to the onset of SAT varied from 5 days [22] to a few years [23, 24]. There have been no reports of SAT associated with other biologic disease-modifying anti-rheumatic drugs (DMARDs) or targeted synthetic DMARDs, such as an IL-6 receptor inhibitor (tocilizumab), CTLA-4 Ig (abatacept), IL-17 inhibitors, IL-23 inhibitors or JAK inhibitors [22]. Moreover, several previous reports demonstrated that IFNα-inhibitor therapy for hepatitis C can also induce SAT [22, 26]. The postulated mechanism has included the hypothesis that TNF inhibition leads to increased production of IFN-α by plasmacytoid dendric cells, which promotes lymphocyte migration and inflammatory reaction [27]. It was also suggested that the mechanism of SAT triggered by TNF inhibitors may be related to cytokine imbalance [22].

## Epidemiology

The highest incidence of SAT regards middle aged women, and females account for 75–80% of all SAT patients [28, 29]. However, the symptoms of SAT were present in as many as 10–20% of patients hospitalized due to COVID-19 on intensive care units (ICU) and non-intensive care units, respectively [18, 30]. Moreover, recently young children affected by SAT have been reported [31, 32]. Thus, the clinicians should be aware of a possible presence of SAT in children, including the youngest ones.

## Current clinical manifestation and factors modifying clinical course

### The hitherto state-of-art

It has been well known for decades that the most common complaints of patients with SAT include anterior neck pain – usually radiating to the jaw, ear and upper mediastinum – as well as fever, rising especially at night. Fatigue, malaise and muscle pain can also occur. Many patients present clinical and/or biochemical manifestation of thyrotoxicosis, with usually low to moderate severity [29]. In the minority of cases, signs and symptoms of thyrotoxicosis may dominate the clinical presentation, with weight loss, tremor and palpitations [29]. The symptoms described above are considered typical for the first phase of SAT. Thyrotoxicosis phase results from the destruction of thyroid follicles and release of thyroid hormones. Typical course includes also the second phase, when pain and fever resolve and the third phase with subsequent hypothyroidism. Persistent hypothyroidism is rare [29].

The most characteristic laboratory finding is a high erythrocyte sedimentation rate (ESR), sometimes reaching even three-digit value. C reactive protein (CRP) is elevated in many cases, although it is a less typical marker. White blood count (WBC) may also be increased. Anti-thyroid antibodies are believed to be usually normal. The ultrasound (US) pattern of SAT include hypoechoic and heterogeneous areas with blurred margins, poorly vascularized on color Doppler [33].

Although the natural course of the disease is believed to be often self-limiting, patients frequently suffer from severe symptoms which persist even for months and not rarely resolve only after glucocorticoid (GC) treatment.

### Novel aspects of the clinical and laboratory manifestation

The clinical characteristics of the disease has been changing significantly for the recent years. More and more cases of painless SAT have been reported [29, 34, 35], with frequency reaching 6.25% in our studies published in 2019 [29], but increasing instantly during the pandemic, including virtually the majority of patients with SAT hospitalized due to COVID-19 [18, 30]. The latter observation was primarily assigned to the frequent application of analgesics and nonsteroidal anti-inflammatory drugs (NSAID) to COVID-19 patients [36]. However, further analyses revealed painless course even in patients with no such treatment [18]. Thus, the neck pain – symptom regarded previously as the key diagnostic criterion – appeared to be not always present.

Fever was also reported to occur less often than it was believed, and was frequently associated with microhaematuria [29]. However, microhaematuria was also observed in some patients with normal body temperature and it resolved spontaneously with SAT resolution [29].

As it was previously mentioned, some cases of children with SAT have been reported [31, 32]. The most spectacular case, published very recently, regarded 5-year-old boy with airway compromise caused by SAT [31]. The boy suffered from fever, painful neck swelling, dysphagia and hoarseness for a few weeks and finally he was admitted to hospital due to dyspnea. US and fine needle aspiration biopsy (FNAB) findings were typical for SAT and magnetic resonance imaging confirmed trachea compression by the enlarged thyroid. Steroid treatment led to symptom resolution [31]. Thus, one should remember about the possibility of SAT occurrence in children, as well as of the fact that symptoms of airway compression may be a manifestation of SAT.

Several years ago, the absence of thyroid antibodies was considered typical for SAT. However, elevated levels of anti-thyroid antibodies, including thyroid peroxidase antibodies (aTPO), thyroglobulin antibodies (aTg), and even thyrotropin receptor antibodies (TRAb) are more often present [29, 37]. The frequency of increased aTPO, aTg and TRAb levels has recently been reported by our research team as 15.52%, 33.33% and 6.0%, respectively [29]. Similar results were reported by Nishikara et al. who observed the presence of aTg and aTPO in 52.5% and 15.6% of samples, respectively [38]. The authors demonstrated that in some patients the aTg concentrations decreased or disappeared within 4 months to 6 years [38]. This observation confirms our previous hypothesis that in many patients the occurrence of thyroid antibodies in SAT may be considered a simple consequence of the release of thyroid antigens due to the gland damage during SAT [29]. However, it can also reflect the increasing incidence of autoimmune thyroid disease (AITD) and the phenomenon of overlapping of SAT and AITD. The crucial significance of co-occurrence of particular HLA alleles and simultaneous presence of SAT and GD has been recently reported in our studies [37].

Despite the progress in availability of diagnostic tools, the differential diagnosis between thyrotoxicosis in GD and SAT may be challenging, especially in resource-limited settings, including undeveloped regions of the world, but also recently due to the limited accessibility of diagnostic tests, caused by COVID-19 pandemic, observed even in some developed countries. Many authors analyzed the usefulness of several surrogate markers based on simple blood count analysis. The potential usefulness of such parameters as platelet-lymphocyte ratio (PLR) [39–42], monocyte-eosinophil ratio (Mo/Eo) combined with free triiodothyronine (FT3) to free thyroxine (FT4) ratio (FT4/FT3) [43], neutrophil–lymphocyte ratio (NLR) [39, 41, 42] has been postulated. Taşkaldiran et al. observed that in Turkish patients with SAT, PLR and NLR were significantly higher than in patients with GD, thyroid adenomas or in healthy control groups [39]. Another study provided similar observation that PLR and NLR values in SAT patients are higher as compared with healthy control subjects [42]. In a large cohort from India, PLR values were also higher in SAT as compared to GD, however, they were significantly lower than in the control euthyroid group [40]. The authors concluded that PLR is a useful marker to differentiate SAT and GD and proposed a PLR cut-off value of 70.4 with demonstrated sensitivity of 86% and specificity of 74% [40]. Cengiz et al. aimed to assess the usefulness of NLR and PLR in the follow up of SAT patients [41]. They proposed cut off values of 2.4 (80% sensitivity and 51% specificity) for NLR and 146.84 (83% sensitivity and 54% specificity) for PLR for the acute phase of the disease. Taking into account the discrepancies in the obtained results, we can conclude that NRL and PLR can be useful in differential diagnosis of SAT and GD and in the follow up of SAT treatment, but currently no universal cut-off value can be unequivocally recommended. Hu et al. evaluated other potential markers and reported that Mo/Eo and FT4/FT3 ratios were significantly higher in SAT than in GD, and the cut-off values for fT4/fT3, Mo/Eo ratios and Mo/Eo ratio + fT4/fT3 for diagnosing GD were ≤ 2.841, ≤ 8.813 and > 0.644, respectively [43]. Although still no universal cut-off values of the proposed markers can be recommended for general population, such surrogate tools might be helpful in the initial differential diagnosis of SAT in resource-limited settings.

It is well known that during SAT, both thyroid lobes or only one lobe may be affected in US. In the latter case, the inflammatory infiltration often migrates from one lobe to the other. However, at the time of diagnosis, the US lesions can usually be described as unilateral or bilateral. Sencar et al. analyzed the association between the degree of thyroid involvement in US (unilateral or bilateral) and the clinical course of SAT [44]. No correlation between the initial thyroid involvement in US and either the risk of recurrence or the risk of permanent hypothyroidism were demonstrated [44]. These findings probably result from the fact that the degree of thyroid involvement in US usually changes with time, and the initial US examination may not reflect the most severe stage of the disease.

### Significance of HLA-background

#### Clinical manifestation

We have recently confirmed the significance of HLA-related background by presenting the example of three siblings with very close onset but different clinical course of SAT [37]. The HLA profile in the reported siblings was strongly correlated with both SAT and GD, however the coexistence of particular sets of high risk and protective alleles seemed to be crucial for the GD development and the SAT course. Neither living in the same environment, nor being fraternal twin sisters was crucial for the clinical manifestation. The patient gender did not seem to be of special importance, either. The only feature directly correlated with the clinical course was the HLA genotype. The co-occurrence of HLA-DRB1*15:01 and/or -B*07:02, possibly together with the lack of HLA-A*01:01 and -B*41:01 seemed to be key factors protecting against the development of GD, as well as against the recurrent SAT course and steroid dependence [37].

#### US pattern

Our research group has recently demonstrated that even the US pattern of SAT thyroid lesions seemed to depend on HLA, and the determining factor was the presence of HLA-B*18:01 [45]. Typically observed multiple hypoechoic blurred lesions were demonstrated to be rarely found in -B*18:01-positive patients. In this group, the US pattern was different and the deviations from the typical SAT US image were mostly pronounced in patients with the presence of HLA-B*18:01 only, without any haplotype correlated with SAT. In most cases with HLA-B*18:01 only, unilateral homogenously hypoechoic single SAT area, filling the whole affected lobe and mimicking the large thyroid nodule was observed. In patients with co-presence of HLA-B*18:01 and -B*35, the main difference from the typical pattern concerned the shape of the SAT lesions, which were patchy or round, imitating actual thyroid nodule [45]. This report explained the phenomenon of various US patterns of SAT. Additionally, the knowledge on the association between HLA and SAT can prevent erroneous exclusion of SAT on the basis of atypical US pattern.

#### Risk of recurrence

The rate of SAT recurrence is rather high, despite a proper diagnosis and treatment, and vary between studies from a few to over 20% [46, 47]. These discrepancies are probably dependent on the studied population (Caucasian vs. Asian). Recurrences of SAT can occur either soon after the completion of treatment or after a significant time interval, sometimes many years from the first episode [47]. Until recently, the cause of SAT recurrences was unknown. It was only postulated that too fast tapering of GC dose is one of the reasons [48]. Recently, we have demonstrated that the risk of SAT recurrence is HLA dependent [47]. Our study, performed in Caucasian population, revealed that the risk of recurrences is significantly higher in patients with co-presence of HLA-B*18:01 and -B*35 [47]. We speculated that it was HLA-B*18:01 that changed the course of SAT, but in the case of recurrence, the co-presence of HLA-B*35 was required and the increased recurrence risk resulted from the combined effect of the two independent key SAT risk haplotypes [47]. The knowledge on this correlation enables better management of high-risk patients. In such cases, the steroid treatment should be intensified with slower dose reduction [47].

As it was discussed above, the risk of SAT recurrence was suggested to be HLA-dependent. Additionally, we have found that biochemical thyrotoxicosis during SAT, with considerably lower thyrotropin (TSH) and higher FT4 and FT3, was significantly more pronounced in patients without recurrences [47]. Higher frequency of elevated concentration of aTPO was also reported in this group. We postulated that increased thyrotoxicosis, resulting from the damage of thyroid follicles, as well as the presence of AITD, are predictive factors of a non-relapse SAT course, and that more severe thyroid tissue damage may play a protective role against recurrences [47]. This observation led to the hypothesis that – perhaps – some AITD-related HLA alleles play a protective role against SAT. Such hypothesis seems possible, taking into account that the most well-known AITD-related antigen – HLA-DRB1*03 – belongs to major histocompatibility complex (MHC) class II while SAT recurrence risk antigens, HLA-B*18:01 and -B*35, belong to MHC class I [47]. Unfortunately, no HLA allele has been so far demonstrated as protective against SAT. Further research is necessary to confirm these hypotheses.

### Significance of a viral factor–SARS-CoV-2

#### The first case report

The first well documented case report of SAT triggered by the SARS-CoV-2 infection was published online in May 2020 [13]. In response to that publication, The Endocrine Society issued a statement on a possible correlation between infection with SARS-CoV-2 and SAT [49]. The authors of the first report described a 18-year-old woman in whom SAT symptoms occurred 15 days after positive PCR test confirming oligosymptomatic SARS-CoV-2 infection. The primary SAT symptoms were similar to the symptoms of infection and included low-grade fever with neck pain, fatigue, and heart palpitations. Laboratory tests revealed features typical for SAT (accelerated ESR, increased CRP, features of thyrotoxicosis). Clinical symptoms resolved a few days after starting CS therapy [13].

#### The time-lag between SARS-CoV-2 infection and SAT symptoms

When the first report was published [13], the 2 week period between positive PCR test and SAT occurrence was considered rather short. However, the further case reports and reports on series of patients have provided surprising observations that SAT symptoms can occur shortly after the beginning of SARS-CoV-2 infection or even co-occur with the symptoms of COVID-19 [36]. Such phenomenon has never been described before in regard to other triggering viral infections. The second reported case described SAT occurrence only 5 days after positive SARS-CoV-2 swab test [36]. Simultaneous presence of SAT and COVID-19 was reported in up to 20% of patients hospitalized due to COVID-19 [18, 30]. Interestingly, in some cases SAT was even the only symptom of the present SARS-CoV-2 infections [50, 51]. San Juan et al. reported a patient with co-presence of fully symptomatic SAT and clinically asymptomatic COVID-19, in whom chest radiograph finally showed right lower lobe pneumonia, although no respiratory symptoms were present [51]. That paper indicates the necessity to perform basic diagnostic procedures of the respiratory tract in a patient with SAT and COVID-19 even if the SARS-CoV-2 infection seems to be clinically asymptomatic.

A typical few weeks period from COVID-19 to SAT occurrence was also described. Ruggeri et al. reported the case of a woman who developed fully symptomatic SAT 6 weeks after upper respiratory tract infection, confirmed as SARS-CoV-2 infection [52]. The time lag between COVID-19 positive PCR swab test and the beginning of SAT do not seem to correlate with COVID-19 severity. Simultaneous SAT was described as the only symptom of COVID-19 [50] and was also reported in patients requiring ICU hospitalization [18]. Similarly, a typical few-week time lag between SARS-CoV-2 infection and SAT was reported in asymptomatic patients [52, 53] as well as in patients after severe course of COVID-19 [54].

#### Clinical symptoms of SAT triggered by COVID-19

As it has been underlined before, neck pain can no longer be considered the main symptom of SAT due to the increasing number of cases of painless SAT course. Such situation requires differential diagnosis with silent thyroiditis [19], which is typically associated with increased aTPO concentration and normal ESR. The higher frequency of painless SAT is evident in the COVID-19 patients. Apart from the fact that neck pain might be assigned to the viral infection itself and simply not reported, there is an increasing number of COVID-19-related painless SAT cases [18, 30, 36]. Actually, at least 3 groups of patients with different reasons of this phenomenon should be identified. The first group includes COVID-19 patients treated with analgesics or NSAIDs. Obviously, many of such patients with SAT during COVID-19 are not diagnosed as SAT because no neck pain is reported. In such patients fever can be also simply assigned to COVID-19. The first case reporting this problem was published in May 2020 [36]. Ippolito et al. described a case of pneumonia in the course of COVID-19 in a patient after back surgery, treated with tramadol, acetaminophen and morphine [36]. No NSAID was administered due to hypersensitivity. On the 5th day after COVID-19 diagnosis the patient developed symptoms of thyrotoxicosis, including palpitations, insomnia and agitation, being the only clinical symptoms of SAT. Thyroid tests revealed decreased TSH with increased FT4 and FT3, and negative thyroid antibodies. Thyroid US pattern was typical for SAT and there was no uptake at thyroid scan using Tc 99-m, which further confirmed the diagnosis. Symptoms subsided after steroid treatment [36]. The second group includes ICU patients who are usually unable to report any pain symptoms because of their general condition and treatment. In a study from a single ICU, none of the SAT patients reported neck pain [18]. Finally, the third group includes patients in whom the SAT course is truly painless. Anyway, the incidence of painless SAT in SARS-CoV-2 positive patients seems to be significantly higher than it was reported in correlation with other triggering viruses. This painless course may be related to reduced lymphocyte-plasmocytic infiltration of thyroid gland, resulting from lymphopenia occurring in COVID-19 patients [16].

Signs and symptoms of thyrotoxicosis used to occur in a minority of SAT patients. However, a presence of tachycardia, a deterioration of tachycardia or an occurrence of heart arrhythmias were reported as typical SAT symptoms in COVID-19 patients [54, 55]. In hospitalized COVID-19 patients, severe tachycardia and episodes of atrial fibrillation occurred in patients with no previous heart disease [54]. This phenomenon probably results from the overlapping effects of biochemical thyrotoxicosis and cytokine storm on the heart function [56]. The severity of thyrotoxicosis was observed to correlate with the concentration of IL-6 [30].

Thyroid tests in SAT patients with severe course of COVID-19 revealed thyrotoxicosis with serum elevated concentration of FT4, low concentrations of TSH and FT3, which corresponds to simultaneous presence of SAT and non-thyroidal illness syndrome in the course of severe systemic disease [18].

#### The most important clinical management recommendations related to SAT triggered by COVID-19

Because of the high frequency of painless SAT in patients hospitalized due to COVID-19, routine assessment of thyroid function should be considered, especially in the ICU group [18], although the World Health Organization (WHO) did not recommend thyroid tests in routine diagnostics of patients with COVID-19 [57]. Specialists working at ICUs should be aware that unexpected deterioration of clinical condition in a patient with COVID-19 may be caused by SAT onset. Gorini et al. shortly summarized the associations between COVID-19 and thyroid disorders and suggested that a simple thyroid test together with the administration of a specific questionnaire at hospital admission with subsequent follow-up could be useful in patients with COVID-19 [58]. The authors underlined two different goals of such approach. Firstly, it seems beneficial because the undiagnosed thyroid dysfunctions – resulting mainly from undetected SAT – may lead to a worse general condition of patients, and – importantly – may alter the metabolism of the drugs used for COVID-19 [58]. Secondly, the data collected through such proceedings will be precious for environmental epidemiology studies [58].

On the other hand, due to the very high frequency of asymptomatic SARS-CoV-2 infections, testing for SARS-CoV-2 infection should be considered in all patients with SAT diagnosed during pandemic. It has to be stressed that SAT might be the only symptom of SARS-CoV-2 infection.

## The most important diagnostic problems

### False negative diagnosis

Signs and symptoms of SAT are non-characteristic. Recent changes in the clinical presentation make the diagnosis even more difficult despite the progress in diagnostic methods. The symptoms of SAT may be assigned to the infection, as neck pain (if present) may be referred to throat or lymph nodes involvement, while fever, fatigue, and increased inflammatory markers (CRP, WBC) may further confirm the false negative diagnosis.

Despite the high availability of diagnostic tools, including laboratory markers and US examination, the diagnosis of SAT is still frequently delayed. The patient often visits many physicians, starting from GPs, through laryngologists and other specialists, before being finally diagnosed with SAT. The period of this delay was reported by our research team as ranging from two weeks even to six months [59]. Due to misdiagnosis of infection, antibiotics were unnecessarily administered in nearly 50% of SAT patients. The ineffectiveness of one antibiotic resulted frequently in the use of another one, covering wider spectrum of bacteria. Characteristic features, occurring more frequently in the antibiotic treated patients included fever, preceding infection, elevated CRP and WBC [59]. These observations are alarming because antibiotic resistance, caused by antibiotic misuse or overuse, is one of the most serious threats to public health and a very important challenge for the current medicine. The WHO, European Commission and health organizations in many countries introduced programs and provided guidelines to prevent antibiotic resistance [60–63]. Despite these recommendations, wide spectrum antibiotics were often administered to SAT patients. Thus, the educational programs, especially directed for GPs should be provided.

For the last year, this issue has been appearing as more important than ever due to COVID-19 pandemic. As it has been described above, the proper SAT diagnosis is even more difficult in patients with COVID-19.

### False positive diagnosis

False negative SAT diagnosis results in a delayed treatment and disturbs patient’s quality of life, although it is not a life-threatening situation. On the other hand, false positive SAT diagnosis can refer to patients with thyroid primary and metastatic malignant tumors of poor prognosis. In 2019, we presented several cases of such malignancies, underlining that the clinical presentation mimicking SAT led to a delayed diagnosis which shorted the patients’ life [64]. The proper diagnosis was made in our Department after—not rarely—many weeks of previous incorrect treatment with NSAIDs and/or steroids administered by GPs or various specialists. Those cases gave us a significant impulse to re-analyze the usefulness of the SAT diagnostic criteria and to modify them adequately to the current state of knowledge. For several decades, SAT diagnosis had been made mostly on the basis of the presence of neck pain and significantly accelerated ESR, which were considered the main diagnostic criteria. These two main criteria plus one of the additional criteria (significantly reduced iodine uptake, transient thyrotoxicosis, fever, typical US pattern, typical FNAB result, low concentration of anti-thyroid antibodies) had been considered enough to diagnose SAT. All of the patients with advanced malignancies, presented in our report, met both the main criteria and one of the additional criteria [64]. Malignant tumors of poor prognosis, such as anaplastic thyroid cancer or progressive neoplasms with thyroid metastases may present with neck pain due to thyroid capsule extension, and elevated ESR due to the advanced progression of cancer. Additionally, thyrotoxicosis can be observed due to the gland destruction, iodine uptake can be decreased and even fever can occur because of the presence of abscess in the necrotic tumor masses. We have reported 5 cases, including 4 anaplastic thyroid carcinomas and one non-small cell lung cancer thyroid metastasis [64]. All of them suffered from neck pain and all had accelerated ESR. The proper diagnosis was made on the basis of atypical US pattern and FNAB results. Thus, we concluded that no SAT diagnosis can be made without US examination [64]. However, one should remember that some US features of malignant lesions, such as blurred lesion margins or hypoechogenity, may be similar to those of SAT. Pan et al. analyzed sonographic features of 165 lesions diagnosed as SAT or malignancies and concluded that useful sonographic predictors of SAT are poorly defined margin, centripetal reduction of echogenicity and the absence of internal vascularity [65]. Nonetheless, a significant overlap of features of SAT and malignant nodules was demonstrated [65]. The possible difficulties in distinguishing SAT and malignancies were also described by other authors [66]. Therefore, if US pattern is doubtful, FNAB should be considered the decisive test [64].

The burning issue of false positive diagnosis of SAT on the basis of neck pain and clinical and biochemical features of inflammation has been raised for many years. Meier and Nagle reported cases of laryngeal carcinoma, Hodgkin’s disease and metastatic lung carcinoma which were initially diagnosed and treated as SAT [67]. Laboratory markers of thyrotoxicosis were present in most of the cases. The final diagnosis of malignancy was made only after the US and FNAB were finally performed due to lack of the treatment effectiveness [67]. Similar situations were reported in the cases of thyroid lymphoma [68], thyroid primary carcinomas, including even follicular carcinoma [69], and thyroid metastases of numerous different malignancies including mainly lung carcinomas [70] and adenocarcinomas of different origins [71, 72]. In all of these cases, the proper diagnosis was possible only on the basis of FNAB result.

Jonklaas has recently analyzed cases of thyrotoxicosis caused by thyroid infiltration by non-thyroid malignancies [73]. This kind of thyroiditis-like presentation was observed mostly in metastases of breast and lung cancers, as well as in hematologic malignancies. The author underlined that the malignancy-associated hyperthyroidism resulted from a destruction of thyroid tissue – a mechanism similar to the one causing thyrotoxicosis in SAT [4].

Therefore, taking into account the large number of malignancies which can mimic SAT, the differential diagnosis based on US should always be introduced, and FNAB should be performed in all doubtful cases.

### Co-presence of SAT and thyroid malignancy

Recent reports have proven that SAT diagnosis is actually not as easy as it was believed before. Malignancies can be erroneously diagnosed as SAT and vice versa. Moreover, one should always remember about rare – but possible – co-presence of SAT and differentiated thyroid carcinoma (DTC). A typical SAT US pattern, being a manifestation of the inflammatory process, does not warrant lack of malignancy. The simultaneous presence of SAT and DTC was believed to be very uncommon and used to be presented mostly as case reports [74, 75]. However, the frequency of co-occurrence of SAT and DTC has recently been proved as higher than it was previously thought. Nishihara et al. conducted a study involving 710 patients with SAT who underwent US examinations at initial diagnosis and during follow-up, with subsequent FNAB evaluation of suspicious nodules [76]. The US examination at the initial screening of thyroid nodules in SAT patients showed a sensitivity of 72.4%, specificity of 89.0%, positive predictive value of 80.4%, and negative predictive value of 83.8%. Co-presence of papillary thyroid carcinoma (PTC) was found in 3.1% of SAT patients. As many as 30% of PTC cases were not identified during the initial scan and were only found during the follow-up US. Interestingly, all tumors in this group were latently localized in the SAT-related bilateral hypoechoic areas of the thyroid and showed no calcifications [76]. On the contrary, out of the 15 tumors detected during the initial examination, 7 presented calcifications and 5 were located in areas unaffected by SAT. The authors underlined that SAT can significantly obscure coexisting PTC when inflammatory hypoechoic regions are present [76]. Gül et al. confirmed the diagnosis of PTC in even higher percentage of SAT patients, reaching 4.4% [77]. Thus, US re-examination after resolution of SAT-related thyroid lesions should always be performed. Any suspicious thyroid lesions – nodules or areas – which do not resolve or which appear after resolution of SAT should undergo FNAB.

## Diagnostic criteria adapted to the current knowledge

Taking into account the observations described above, in 2019 we proposed the new SAT diagnosis criteria [64]. After a year of COVID-19 pandemic we believe that those criteria are getting more and more actual due to significant frequency of painless SAT and several novel aspects of clinical course of SAT triggered by SARS-CoV-2 infection. We postulated that in order to undoubtedly diagnose SAT, all of the 2 main criteria should be met, together with at least one of the additional criteria [64]. These criteria, supplemented by COVID-19-related remarks, are presented in Table 1.

**Table 1:** Diagnostic criteria of subacute thyroiditis (based on [64], modified)

Main criteria (all should be met)	Additional criteria (at least one should be met):
1. Laboratory: elevation of ESR or at least CRP 2. Ultrasound: hypoechoic area/areas with blurred margin and decreased vascularization in US*	1. Hard thyroid swelling 2. Pain and tenderness of the thyroid gland/lobe 3. Elevation of serum FT4 and suppression of TSH 4. Decreased radioiodine uptake 5. FNAB result typical for SAT
*FNAB should be performed in all doubtful cases and in patients that show no improvement on a short term follow-up, in order to exclude malignancy	
Remarks related to COVID-19 pandemic (should be taken into account during pandemic)	
1. SAT diagnosis should be considered in patients with/after SARS-CoV-2 infections with: 1.1 unexpected: 1.1.1 *de novo* presence of tachycardia or arrhythmias 1.1.2 deterioration of previously present tachycardia or arrhythmias 1.1.3 deterioration of fatigue/malaise 1.2 laboratory markers of thyrotoxicosis, including decreased TSH and increased FT4 – thyroid tests should be considered in all patients hospitalized due to COVID-19, especially in ICU patients	
2. SAT is more frequently painless in COVID-19 patients and the presence of pain should not be treated as SAT criterion in this group, especially in hospitalized patients	
3. As SAT may be the only manifestation of COVID-19, testing for SARS-CoV2 infection should be considered in all patients with SAT diagnosed during the pandemic	

## Novel aspects of the treatment

Recurrent course of SAT and steroid dependence are important problems of SAT treatment. It is difficult to find balance between the risk of recurrence during GC dose tapering and the risk of complications of long-term GC therapy. Tian et al. described three patients with SAT recurrences after a reduction in prednisolone (PSL) dose, either immediately upon the cessation of PSL or shortly thereafter [78]. These patients were successfully treated with colchicine at 1 mg per day for 1–2 months, either together with PSL (2 cases) or alone (1 case) [78]. The potential risk of colchicine toxicity associated with the chronic administration should be carefully considered. Large-scale, controlled, preferably blinded studies are required to assess efficacy and safety of such therapy. Therefore, the treatment with colchicine can be carefully considered only in patients with steroid dependent SAT with multiple recurrences as a potential thyroid-saving therapy in the cases with GC resistance.

Interesting results of a randomized controlled study were reported by Duan et al. who compared results of treatment of patients with moderate-to-severe SAT symptoms with either 30 mg/d PSL for 1 week, followed by 1 week of NSAID, or 6-week PSL therapy [79]. Fewer side effects of GC and similar efficacy and recurrence rates were observed in patients with short-term PSL as compared with the 6-week treatment [79]. The results are highly surprising, as it is well known that too short steroid treatment is associated with significant risk of recurrence [47, 48]. The study group and control group included 26 and 24 patients, respectively. The recurrence rates were substantially high in both groups, reaching 30.8% and 25.0%, respectively [79]. Whether these numbers resulted from the relatively small number of patients or from some factors related to the high overall risk of recurrence, requires further investigations. One should take into account that the prolongation of treatment in the control group to 8–10 weeks would have potentially resulted in a reduced number of recurrences, and a disclosure of significant differences between the groups. Moreover, as the risk of recurrence may be HLA-dependent, it should be considered that both groups were recruited in the same demographic area, so the influence of HLA in such group might have been exceptionally strong. The results of the study should lead to further similar trials, with higher number of patients, to assess how to avoid prolonged steroid treatment. However, the proposed short-term strategy is rather difficult to be currently recommended due to the high rate of recurrence.

## Summary

A few recent years have brought unprecedented progress in the current knowledge on SAT pathogenesis, epidemiology, clinical course and management. The summary of the most important advances in SAT and their clinical implications is presented in Table 2. Clinical course of SAT was demonstrated to be dependent on genetic background, as well as on the triggering factors – especially in regard to SARS-CoV-2 infection. It has been proved for the first time that there is a close relationship between the course of SAT and the specific sets of HLA alleles carried by a given patient. Additionally, the example of COVID-19 has pointed out the spectacular importance of this triggering factor on the SAT course, bringing the hypothesis that other viral and non-viral triggers may also modify the SAT course. In the era of immense progress in targeted therapies, TNF-α inhibitors should also be considered as potential SAT triggering factors and similar properties of other novel therapies require further elucidation.

**Table 2:** Summary of the new findings in SAT and their clinical implications

	Novel findings	Clinical implication
Genetic background	High risk alleles [10]:	Testing towards these four alleles can provide information about the SAT susceptibility (data for Caucasian population) [10]
	• HLA-B*35:01/02/03	
	• HLA-C*04:01	
	• HLA-B*18:01	
	• HLA-DRB1*01	
Triggering factors	1. Viral: • SARS-CoV-2 [49]	1. SARS-CoV-2 infection may trigger unexpected number of SAT cases – medical care providers should be aware of such possibility and of differences in SAT clinical course
	2. Non-viral: • TNFα-inhibitors (confirmed for adalimumab, etanercept, infliximab) [22–26]	2. Medical stuff should inform patients about the risk and about signs and symptoms of SAT
Epidemiology	1. High incidence in COVID-19 hospitalized patients (10–20%) [18, 30]	1. Consider thyroid hormone testing and/or other SAT screening methods especially in ICU patients
	2. Possible occurrence in young children [31, 32]	2. Physicians should be aware about possible SAT occurrence in children; possible atypical course with trachea compression [31]
Clinical course	1. More frequent painless course: • >6% in Caucasian population [29]	1. Neck pain cannot be considered the main symptom or main diagnostic SAT criterion
	• majority of COVID-19 hospitalized patients [18, 30]	
	2. Possible presence of aTPO, aTg or even TRAb [29]	2. The presence of thyroid antibodies cannot exclude SAT and can be transient or persistent [29]; coincidence with GD is possible in patients with HLA-related susceptibility [37]
HLA correlations with clinical course	1. Clinical manifestation of SAT in patients with co-presence of GD depends on constellation of specific HLA alleles [37]	Clinicians should be aware of these correlations to properly conduct the diagnostic process
	2. US pattern depends on the presence of HLA-B*18:01 [45]	
	3. Risk of SAT recurrence depends on the co-presence of HLA-B*18:01 and -B*35 [47]	
Clinical course of SAT triggered by COVID-19	1. SAT is common in COVID-19 hospitalized patients (up to 10–20%) [18, 30]	1. Thyroid hormone testing should be considered in COVID-19 hospitalized patients
	2. SAT onset may occur simultaneously with SARS-CoV-2 infection [18, 30, 36, 50, 51]	2. If any symptoms are present, SAT should be suspected even during a current active COVID-19
	3. Very frequent painless course, occurring in most of hospitalized COVID-19 patients [18, 30]	3. Pain is not a diagnostic criterion in COVID-19 patients, especially in hospitalized ones
	4. Tachycardia/arrhythmias and/or malaise/fatigue may be the only SAT symptoms [54, 55]	4. In COVID-19 patients with tachycardia/ arrhythmias or unexpected deterioration of clinical condition, SAT should be suspected
	5. Low TSH and FT3 with elevated FT4 are typical for SAT in COVID-19 patients hospitalized in ICU [18], low TSH with high FT3 and FT4 are typical for SAT in patients with less severe COVID-19	5. Thyroid tests should be performed in COVID-19 patients with SAT-like symptoms and should be considered in all hospitalized patients, especially in ICU
	6. SAT may be the only manifestation of SARS-CoV-2 infection [50]	6. Testing for SARS-CoV-2 infection should be considered in all patients with SAT diagnosed during pandemic
Malignancy-related diagnostic problems	1. Primary and metastatic thyroid tumours may mimic SAT [64–72]	1. US is mandatory for SAT diagnosis, FNAB recommended in all doubtful cases [64]
	2. Possible co-presence of DTC – tumours not visible in US due to SAT inflammatory lesions [76, 77]	2. US monitoring of SAT patients is required

The differential diagnosis of SAT is still challenging. The significant concern is to avoid false negative and – more importantly – false positive SAT diagnoses, as such situations pose a significant risk to patients, especially in regard to undiagnosed malignancies. We believe that application of the new diagnostic criteria proposed in our recent report [62] and presented above in the modified version complemented with novel aspects related to COVID-19 pandemic (Table 1), will facilitate and speed up the differential diagnosis of SAT.

## Supplementary Information

Below is the link to the electronic supplementary material.

Supplementary file1 (PDF 275 KB)

## Abbreviations

ACE2: Angiotensin converting enzyme 2
ADA: Adalimumab
AITD: Autoimmune thyroid disease
aTg: Thyroglobulin antibodies
aTPO: Thyroid peroxidase antibodies
CRP: C reactive protein
COVID-19: Coronavirus disease 19
DMARDs: Disease-modifying anti-rheumatic drugs
DTC: Differentiated thyroid carcinoma
ESR: Erythrocyte sedimentation rate
FNAB: Fine needle aspiration biopsy
FT3: Free triiodothyronine
FT4: Free thyroxine
GD: Graves’ disease
HLA: Human leukocyte antigens
GC: Glucocorticoid
ICU: Intensive care unit
IL: Interleukin
MHC: Major histocompatibility complex
NSAID: Nonsteroidal anti-inflammatory drug
PSL: Prednisolone
PTC: Papillary thyroid carcinoma
SARS: Severe acute respiratory syndrome
SARS-CoV-2: Severe acute respiratory syndrome-coronavirus-2
SAT: Subacute thyroiditis
TNF: Tumor necrosis factor
TRAb: Thyrotropin receptor antibodies
TSH: Thyrotropin
US: Ultrasound
WBC: White blood count
WHO: World Health Organization

## References

[1] Nyulassy S, Hnilica P, Stefanovic J (1975). The HLA system and subacute thyroiditis. A preliminary report Tissue Antigens.

[2] Nyulassy S, Hnilica P, Buc M, Guman M, Hirschová V, Stefanovic J (1977). Subacute (de Quervain’s) thyroiditis: Association with HLA-Bw35 antigen and abnormalities of the complement system, immunoglobulins and other serum proteins. J Clin Endocrinol Metab.

[3] Yeo PP, Chan SH, Aw TC, Lui KF, Rauff, Mathew T, et al. HLA and Chinese patients with subacute (De Quervain’s) thyroiditis. Tissue Antigens 1981;17:249–50. 10.1111/j.1399-0039.1981.tb00694.x. 10.1111/j.1399-0039.1981.tb00694.x 7233421

[4] Goto H, Uno H, Tamai H, Kuma K, Hayashi Y, Matsubayashi S (1985). Genetic analysis of subacute (de Quervain’s) thyroiditis. Tissue Antigens.

[5] Kramer AB, Roozendaal C, Dullaart RP (2004). Familial occurrence of subacute thyroiditis associated with human leukocyte antigen-B35. Thyroid.

[6] Zein EF, Karaa SE, Megarbane A (2007). Familial occurrence of painful subacute thyroiditis associated with human leukocyte antigen-B35. Presse Med.

[7] Ohsako N, Tamai H, Sudo T, Mukuta T, Tanaka H, Kuma K (1995). Clinical characteristics of subacute thyroiditis classified according to human leukocyte antigen typing. J Clin Endocrinol Metab.

[8] Kobayashi N, Tamai H, Nagai K, Matsubayashi S, Matsuzuka F, Kuma K (1985). Studies on the pathogenesis of subacute thyroiditis. Nihon Naibunpi Gakkai Zasshi.

[9] Buc M, Nyulassy S, Hnilica P, Busová B, Stefanovic J (1979). The frequency of HLA-Dw1 determinant in subacute (de Quervain’s) thyroiditis. Tissue Antigens.

[10] Stasiak M, Tymoniuk B, Michalak R, Stasiak B, Lewiński A (2020). Subacute thyroiditis is associated with *HLA-B*18:01*, *-DRB1*01* and *-C*04:01* – the significance of the new molecular background. J Clin Med.

[11] Available online: www.ctht.info/Table%209%20CB%20ASSOCIATIONS.pdf (Accessed on 20 Nov 2020)

[12] Desailloud R, Hober D, Virol J (2009). Viruses and thyroiditis: an update. Virol J.

[13] Brancatella A, Ricci D, Viola N, Sgrò D, Santini F, Latrofa F (2020). Subacute thyroiditis after SARS-CoV-2 infection. J Clin Endocrinol Metab.

[14] Mangaraj S (2020). Subacute thyroiditis complicating dengue fever - Case report and brief review of literature. Trop Doct.

[15] Guan WJ, Ni ZY, Hu Y, Liang WH, Ou CQ, He JX (2020). Clinical characteristics of coronavirus disease 2019 in China. N Engl J Med.

[16] Caron P (2020). Thyroid disorders and SARS-CoV-2 infection: from pathophysiological mechanism to patient management. Ann Endocrinol (Paris).

[17] Li MY, Li L, Zhang Y, Wang XS (2020). Expression of the SARS-CoV-2 cell receptor gene ACE2 in a wide variety of human tissues. Infect Dis Poverty.

[18] Muller I, Cannavaro D, Dazzi D, Covelli D, Mantovani G, Muscatello A (2020). SARS-CoV-2-related atypical thyroiditis. Lancet Diabet Endocrinol.

[19] Scappaticcio L, Pitoia F, Esposito K, Piccardo A, Trimboli P (2020). Impact of COVID-19 on the thyroid gland: an update. Rev Endocr Metab Disord.

[20] Rotondi M, Coperchini F, Ricci G, Denegri M, Croce L, Ngnitejeu ST (2020). Detection of SARS-COV-2 receptor ACE-2 mRNA in thyroid cells: a clue for COVID-19-related subacute thyroiditis. J Endocrinol Invest.

[21] Mateu-Salat M, Urgell E, Chico A (2020). SARS-CoV-2 as a trigger for autoimmune disease: report of two cases of Graves’ disease after COVID-19. J Endocrinol Invest.

[22] Nakagawa J, Fujikawa K, Akagi M, Nakaji K, Yasui J, Hanatani Y (2021). Subacute thyroiditis in a patient with psoriatic arthritis switched from secukinumab to adalimumab: a case report and literature review. Mod Rheumatol Case Rep.

[23] Yasuji I (2013). Subacute thyroiditis in a patient with juvenile idiopathic arthritis undergoing etanercept treatment: a case report and review of the literature. Mod Rheumatol.

[24] Pascart T, Ducoulombier V, Roquette D, Perimenis P, Coquerelle P, Maury F (2014). Autoimmune thyroid disorders during anti-TNFalpha therapy: Coincidence, paradoxical event or marker of immunogenicity?. Joint Bone Spine.

[25] Kawashima J, Naoe H, Sasaki Y, Araki E (2015). A rare case showing subacute thyroiditis-like symptoms with amyloid goiter after anti-tumor necrosis factor therapy. Endocrinol Diabetes Metab Case Rep.

[26] Shen L, Bui C, Mansberg R, Nguyen D, Alam-Fotias D (2005). Thyroid dysfunction during interferon alpha therapy for chronic hepatitis C. Clin Nucl Med.

[27] Seneschal J, Milpied B, Vergier B, Lepreux S, Schaeverbeke T, Taïeb A (2009). Cytokine imbalance with increased production of interferon-alpha in psoriasiform eruptions associated with anti-tumour necrosis factor-alpha treatments. Br J Dermatol.

[28] Samuels MH (2012). Subacute, silent, and postpartum thyroiditis. Med Clin North Am.

[29] Stasiak M, Michalak R, Stasiak B, Lewiński A (2019). Clinical characteristics of subacute thyroiditis is different than it used to be – current state based on 15 years own material. Neuro Endocrinol Lett.

[30] Lania A, Sandri MT, Cellini M, Mirani M, Lavezzi E, Mazziotti G (2020). Thyrotoxicosis in patients with COVID-19: the THYRCOV study. Eur J Endocrinol.

[31] Ramineni P, Kamath SP, Joshi J, Rao S (2020). Subacute thyroiditis with airway compromise in a 5-year-old boy. BMJ Case Rep.

[32] Bilbao NA, Kaulfers AD, Bhowmick SK (2019). Subacute thyroiditis in a child. Clin Case Rep.

[33] Vural Ç, Paksoy N, Gök ND, Yazal K (2015). Subacute granulomatous (De Quervain’s) thyroiditis: Fine-needle aspiration cytology and ultrasonographic characteristics of 21 cases. Cytojournal.

[34] Al-Tikrity MA, Magdi M, Abou Samra AB, Elzouki AY (2020). Subacute thyroiditis: an unusual presentation of fever of unknown origin following upper respiratory tract infection. Am J Case Rep.

[35] Anyfantakis D, Katsanikaki F, Kastanakis S (2020). An elderly woman with pyrexia of unknown origin. Maedica (Bucur).

[36] Ippolito S, Dentali F, Tanda ML (2020). SARS-CoV-2: a potential trigger for subacute thyroiditis? Insights from a case report. J Endocrinol Invest.

[37] Stasiak M, Lewiński A (2020). Strong correlation between HLA and clinical course of subacute thyroiditis-a report of the three siblings. Genes (Basel).

[38] Nishihara E, Amino N, Kudo T, Kohsaka K, Ito M, Fukata S (2019). Moderate Frequency of Anti-Thyroglobulin Antibodies in the Early Phase of Subacute Thyroiditis. Eur Thyroid J.

[39] Taşkaldiran I, Omma T, Önder ÇE, Firat SN, Koç G, Kiliç MK (2019). Neutrophil-to-lymphocyte ratio, monocyte-to-lymphocyte ratio, and platelet-tolymphocyte ratio in different etiological causes of thyrotoxicosis. Turk J Med Sci.

[40] Dasgupta R, Atri A, Jebasingh F, Hepzhibah J, Christudoss P, Asha HS, et al. Platelet-lymphocyte ratio (PLR) as a novel surrogate marker to differentiate thyrotoxic patients with Graves' disease (GD) from subacute thyroiditis (SAT): a cross-sectional study from South India. Endocr Pract. 2020;14. 10.4158/EP-2020-0086. 10.4158/EP-2020-0086 33471697

[41] Cengiz H, Varim C, Demirci T, Cetin S (2020). Hemogram parameters in the patients with subacute thyroiditis. Pak J Med Sci.

[42] Calapkulu M, Sencar ME, Sakiz D, Duger H, Ozturk Unsal I, Ozbek M (2020). The prognostic and diagnostic use of hematological parameters in subacute thyroiditis patients. Endocrine.

[43] Hu Y, Zhou D, Chen J, Shan P (2020). Eosinophil/monocyte ratio combined with serum thyroid hormone for distinguishing Graves' disease and subacute thyroiditis. Front Endocrinol (Lausanne).

[44] Sencar ME, Calapkulu M, Sakiz D, Akhanli P, Hepsen S, Duger H (2020). The contribution of ultrasonographic findings to the prognosis of subacute thyroiditis. Arch Endocrinol Metab.

[45] Stasiak M, Tymoniuk B, Adamczewski Z, Stasiak B, Lewiński A (2019). Sonographic pattern of subacute thyroiditis is HLA-dependent. Front Endocrinol (Lausanne).

[46] Mizukoshi T, Noguchi S, Murakami T, Futata T, Yamashita H (2001). Evaluation of recurrence in 36 subacute thyroiditis patients managed with prednisolone. Intern Med.

[47] Stasiak M, Tymoniuk B, Stasiak B, Lewiński A (2019). The Risk of Recurrence of Subacute Thyroiditis Is HLA-Dependent. Int J Mol Sci.

[48] Arao T, Okada Y, Torimoto K, Kurozumi A, Narisawa M, Yamamoto S (2015). Prednisolone dosing regimen for treatment of subacute thyroiditis. J UOEH.

[49] Patients with COVID-19 may develop thyroid infection. 21.05.2020. https://www.endocrine.org/newsandadvocacy/newsroom/2020/patientswithcovid19maydevelopthyroidinfection (Accessed: 20 Oct 2020).

[50] Asfuroglu Kalkan E, Ates I (2020). A case of subacute thyroiditis associated with COVID-19 infection. J Endocrinol Invest.

[51] San Juan MDJ, Florencio MQV, Joven MH (2020). Subacute thyroiditis in a patient with coronavirus disease 2019. AACE Clin Case Rep.

[52] Ruggeri RM, Campennì A, Siracusa M, Frazzetto G, Gulloet D (2020). Subacute thyroiditis in a patient infected with SARS-CoV-2: an endocrine complication linked to the COVID-19 pandemic. Hormones (Athens).

[53] Campos-Barrera E, Alvarez-Cisneros T, Davalos-Fuentes M (2020). Subacute thyroiditis associated with COVID-19. Case Rep Endocrinol.

[54] Brancatella A, Ricci D, Cappellani D, Viola N, Sgrò D, Santiniet F (2020). Is subacute thyroiditis an underestimated manifestation of SARS-CoV-2 infection? Insights from a case series. J Clin Endocrinol Metab.

[55] Mattar SAM, Koh SJQ, Rama Chandran S, Cherng BPZ (2020). Subacute thyroiditis associated with COVID-19. BMJ Case Rep.

[56] Dhakal BP, Sweitzer NK, Indik JH, Acharya D, William P (2020). SARS-CoV-2 infection and cardiovascular disease: COVID-19 heart. Heart Lung Circ.

[57] World Health Organization. Clinical management of severe acute respiratory infection (SARI) when COVID-19 disease is suspected: Interim guidance 13 March 2020. https://www.who.int/docs/defaultsource/coronaviruse/clinicalmanagementofnovelcov.pdf

[58] Gorini F, Bianchi F, Iervasi G (2020). COVID-19 and Thyroid: Progress and Prospects. Int J Environ Res Public Health.

[59] Stasiak M, Michalak R, Stasiak B, Lewiński A (2020). time-lag between symptom onset and diagnosis of subacute thyroiditis - how to avoid the delay of diagnosis and unnecessary overuse of antibiotics. Horm Metab Res.

[60] Antimicrobial resistance: global report on surveillance. WHO 2014. www.who.int

[61] Global action plan on antimicrobial resistance. WHO 2015. www.who.int.

[62] European One Health Action Plan against Antimicrobial Resistance (AMR). European Commission. 2017. https://ec.europa.eu/health/amr/sites/amr/files/amr_action_plan_2017_en.pdf (Accessed 10 Dec 2020).

[63] https://www.ecdc.europa.eu/en/publications-data/antibiotic-resistance-policy-briefing-design-files (Accessed 10 Dec 2020).

[64] Stasiak M, Michalak R, Lewinski A (2019). Thyroid primary and metastatic malignant tumours of poor prognosis may mimic subacute thyroiditis - time to change the diagnostic criteria: case reports and a review of the literature. BMC Endocr Disord.

[65] Pan FS, Wang W, Wang Y, Xu M, Liang JY, Zheng YL (2015). Sonographic features of thyroid nodules that may help distinguish clinically atypical subacute thyroiditis from thyroid malignancy. J Ultrasound Med.

[66] Park SY, Kim EK, Kim MJ, Kim BM, Oh KK, Hong SW (2006). Ultrasonographic characteristics of subacute granulomatous thyroiditis. Korean J Radiol.

[67] Meier DA, Nagle CE (1996). Differential diagnosis of a tender goiter. J Nucl Med.

[68] Gochu J, Piper B, Montana J, Park HS, Poretsky L (1994). Lymphoma of the thyroid mimicking thyroiditis in a patient with the acquired immune deficiency syndrome. J Endocrinol Investig.

[69] Prakash R, Jayaram G, Singh RP. Follicular thyroid carcinoma masquerading as subacute thyroiditis. Diagnosis using ultrasonography and radionuclide thyroid angiography. Australas Radiol. 1991;35:174–7. 10.1111/j.1440-1673.1991.tb02860.x 1930018

[70] Shirahama T, Ashitani J, Kodama T, Kyoraku Y, Sano A, Matsumoto N (2008). A case of lung cancer with hyperthyroidism. Nihon Kokyuki Gakkai Zasshi.

[71] Watts NB, Sewell CW (1988). Carcinomatous involvement of the thyroid presenting as subacute thyroiditis. Am J Med Sci.

[72] Eriksson M, Ajmani SK, Mallette LE (1977). Hyperthyroidism from thyroid metastasis of pancreatic adenocarcinoma. JAMA.

[73] Jonklaas J (2020). Infiltration of the thyroid gland by non-thyroid malignancy: A literature review reveals this to be an unusual cause of hyperthyroidism. J Clin Transl Endocrinol.

[74] Şenel F, Karaman H, Ertan T (2016). Co-occurrence of subacute granulomatous thyroiditis and papillary microcarcinoma. Kulak Burun Bogaz Ihtis Derg.

[75] Ucan B, Delibasi T, Cakal E, Arslan MS, Bozkurt NC, Demirci T, Ozbek M, Sahin M (2014). Papillary thyroid cancer case masked by subacute thyroiditis. Arq Bras Endocrinol Metabol.

[76] Nishihara E, Kudo T, Ito M, Fukata S, Nishikawa M, Nakamura H (2020). Papillary thyroid carcinomas are highly obscured by inflammatory hypoechoic regions caused by subacute thyroiditis: a longitudinal evaluation of 710 patients using ultrasonography. Endocr J.

[77] Gül N, Üzüm AK, Selçukbiricik ÖS, Yegen G, Tanakol R, Aral F (2018). Prevalence of papillary thyroid cancer in subacute thyroiditis patients may be higher than it is presumed: retrospective analysis of 137 patients. Radiol Oncol.

[78] Tian Z, Su Y, Zhang M, Zhang X, Guan Q (2020). Successful management of recurrent subacute thyroiditis by adding colchicine to glucocorticoid treatment: a case series study. Horm Metab Res.

[79] Duan L, Feng X, Zhang R, Tan X, Xiang X, Shen R (2020). Short-term versus 6-week prednisone in the treatment of subacute thyroiditis: a randomized controlled trial. Endocr Pract.

