# Supplementary material for: New aspects in the pathogenesis and management of subacute thyroiditis
Source: Rev Endocr Metab Disord. 2021 May 5;22(4):1027–39. doi: 10.1007/s11154-021-09648-y (PMC8096888; doi:10.1007/s11154-021-09648-y)
Supplement: Supplementary file 1 — Supplementary file1 (PDF 275 KB) [file 11154_2021_9648_MOESM1_ESM.pdf]

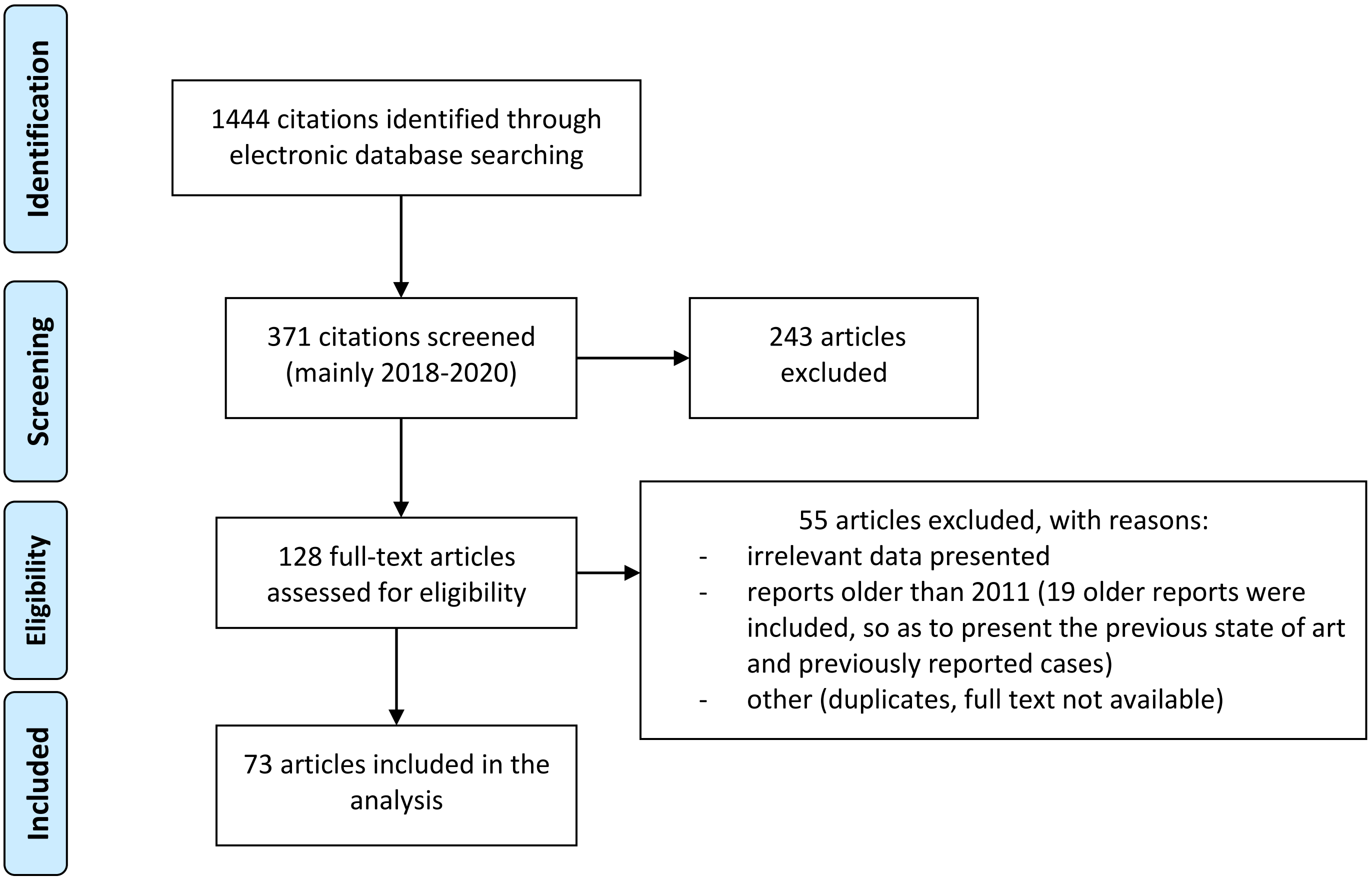

Figure S1. Study selection flowchart outlining the protocol adopted in this review based on the Preferred Reporting Items for Systematic Reviews and Meta-Analyses (PRISMA) Four-Phase Flow Diagram.
